# The Antibacterial Activity of Egyptian Wasp Chitosan-Based Nanoparticles against Important Antibiotic-Resistant Pathogens

**DOI:** 10.3390/molecules27217189

**Published:** 2022-10-24

**Authors:** Eman E. Essa, Dalia Hamza, Mostafa M. H. Khalil, Hala Zaher, Dina Salah, Ashwaq M. Alnemari, Magda H. Rady, Shimaa A. A. Mo`men

**Affiliations:** 1 Entomology Department, Faculty of Science, Ain Shams University, Cairo 11566, Egypt; 2 Department of Zoonoses, Faculty of Veterinary Medicine, Cairo University, Giza 11221, Egypt; 3 Chemistry Department, Faculty of Science, Ain Shams University, Cairo 11566, Egypt; 4 Biophysics Group, Physics Department, Faculty of Science, Ain Shams University, Cairo 11566, Egypt; 5 Biology Department, College of Science and Humanities, Prince Sattam bin Abdulaziz University, P.O. Box 83, Al-Kharj 11940, Saudi Arabia

**Keywords:** vespa orientalis, wasp chitosan nanoparticles, ESBL-, carbapenemase-producing bacteria, antibacterial property

## Abstract

The current work discusses the production and characterization of new biodegradable nanoparticles for biomedical applications based on insect chitosan. Chitosan has numerous features due to the presence of primary amine groups in repeating units, such as antibacterial and anticancer activities. When polyanion tripolyphosphate is added to chitosan, it creates nanoparticles with higher antibacterial activity than the original chitosan. In this study, the ionic gelation technique was used to make wasp chitosan nanoparticles (WCSNPs) in which TEM and FTIR were used to investigate the physicochemical properties of the nanoparticles. In addition, the antibacterial activities of chitosan nanoparticles against extended-spectrum beta-lactamase (ESBL)- and carbapenemase-producing *Klebsiella pneumoniae*, *Escherichia coli*, and *Pseudomonas aeruginosa* were evaluated. The extracted wasp chitosan exhibited high solubility in acetic acid and met all standard criteria of all characterization testes for nanoparticles; the zeta potential indicated stable WCSNPs capable of binding to cellular membrane and increasing the cellular uptake. The produced WCSNPs showed growth inhibition activity against all tested strains, and the bacterial count was lower than the initial count. The inhibition percent of WCSNPs showed that the lowest concentration of WCSNPs was found to be effective against tested strains. WCSNPs’ antibacterial activity implies that they could be used as novel, highly effective antibacterial agents in a variety of biological applications requiring antibacterial characteristics.

## 1. Introduction

Multidrug resistance (MDR) is a global public health issue worldwide where treatment of multidrug-resistant bacterial pathogens with currently available antibiotics becomes challenging [1]. Nowadays, there is a frequent focus on extended-spectrum beta-lactamase (ESBL)- and carbapenemase-producing *Enterobacteriaceae* in humans and animals [2]. MDR microorganisms gain resistance to β-lactam antibiotics by generating plasmid-mediated β-lactamases to oxyimino-cephalosporins including cefotaxime, ceftriaxone, ceftazidime, and monobactams [3]. Notably, MDR zoonotic pathogens *P. aeruginosa*, *K. pneumoniae*, and *E. coli* that produce ESBLs represent a great concern for scientists to tackle such pathogens [4]. Thus, new medicines and innovative chemicals to attack these infections without acquiring resistance are highly needed [5], so we must develop new antibacterial drugs that are effective against the ever-increasing number of antibiotic-resistant pathogenic bacteria [6].

Insects are presently receiving lots of attention as a new source of important and beneficial compounds including lipids, (antimicrobial) peptides, polymers, proteins, and vitamins for a variety of uses [7,8,9,10,11]. Chitin has become more common among natural polymers as it may form up to 25–60% of the dry weight of an insect’s cuticle, and it is embedded in a sclerotized protein matrix with lipoproteins and other components. Moreover, chitin is found in the cuticle’s procuticle (deepest layer) as nanofibers organized in fiber bundles, resulting in an asymmetrical sheet structure that gives the cuticle its elasticity and stiffness [12]. The use of insects in the synthesis of high-value chemicals, and their ease of reproduction on many natural substrates have led to a massive increase in farming [13,14].

Dead wasps of the species *Vespa orientalis* are the sole by-product of a zero-waste method in which the chitin-rich insect waste biomass is gathered around bee colonies after sting removal. This biomass might be a valuable source of chitin as well as its polymers, making insect breeding a totally circular process with a positive economic impact. Although research on insect chitin and chitosan is still in its early stages for many applications, the scientific community is making significant attempts to validate the use of this polymer and its derivatives in a variety of sectors. There are current efforts targeted at collecting chitin from insects to create nanofibers for application in the biomedical and cosmetic industries [14].

The most common chitin derivative is chitosan (CS), which is generated by deacetylating chitin (i.e., partial removal of the acetyl groups from the polymer chain). Chitosan considerably widens the spectrum of chitin uses due to its increased amount of free amino groups, which makes it much more soluble and reactive [15]. Due to the crystalline structure of chitin, the use of deacetylases in enzymatic deacetylation may not always result in effective deacetylation. Consequently, the most common method for producing chitosan from crustaceans and insects is chemical deacetylation using highly concentrated sodium hydroxide solutions at high temperatures [16]. Notably, chitosan is gaining importance as an antibacterial agent since bacteria are not reported to develop resistance to it [17].

Chitosan nanoparticles (CSNPs) were first produced in 1994 by Ohya and coworkers [18]. Many researchers have shown that chitosan nanoparticles and their derivatives have antibacterial properties; however, evidence concerning insect-based chitosan nanoparticles is rather limited [19,20,21]. This is the first study on the in vitro antibacterial properties of WCNPs, which we produced and described using the insect *Vespa orientalis* against antibiotic resistant bacteria of public health impact.

The aim of this research is to support the possibility for insect-based chitosan nanoparticles (wasp chitosan nanoparticles) to be employed as an antibacterial agent in well-established commercial polymer applications.

## 2. Experimental Procedures

### 2.1. Extraction of Wasp Chitosan (WCH)

The dead wasps, *Vespa orientalis*, were collected after sting removal in large masses from the electrical shock chambers, designed to extract wasp venom, which were placed near honeybee colonies to hunt parasitic wasps in the Apiculture Research Department, Dokki, Egypt. The chitin was extracted first following the standard procedure mentioned by [8]. The wasp mass was washed several times with distilled water, dried at 50 °C overnight in an incubator/ drying oven, and ground in the electric mixer. The raw material (wasp powder) was treated with 1.0 M HCl solution to a solid ratio of 15 mL/g at 100°C for 20 min for demineralization. The resultant solid fraction was rinsed with distilled water until it attained a neutral pH value. The following step, deproteinization, was performed using 1.0 M sodium hydroxide at 85 °C. This alkaline treatment was repeated four times over 24 h and rinsed with distilled water until reaching a neutral pH value, then a mild oxidizing treatment (H_2_O_2_/33% HCl 9:1, v:v) was used to remove pigment traces responsible for the brown color of the product [22]. Finally, the obtained lightly brown chitin was washed with distilled water and dried in an incubator/drying oven at 50 °C. The extracted chitin was then rinsed with distilled water until neutralization. The wasp chitin was treated with 50% NaOH at 100 °C for 2 h on a hot plate for deacetylation. The mixture was stirred thoroughly each time for a homogenous reaction. Samples were then cooled, and the resulting chitosan was washed continuously with 50% NaOH and filtered to retain the solid matter, which was further rinsed with deionized water and dried in the oven at 105 °C for 24 h. The previous deacetylation step was repeated for additional periods as an attempt to improve solubility; the time required for this step differs according to the solubility degree of the produced chitosan. Deacetylation of chitin into chitosan was confirmed using the chitin /chitosan color change confirmatory test [23]. The degree of deacetylation was determined and found to be 92–93% [8].

### 2.2. Characterization of Wasp Chitosan (WCS)

Extracted wasp chitosan (WCS) was tested for solubility in dilute acetic acid before being characterized using Fourier transform infrared spectroscopy (FT-IR), in which transmission spectra of the extracted wasp chitosan were compared to the standard chitosan, and the molecular weight of the chitosan polymer was determined via viscosity measurement.

#### 2.2.1. Fourier Transform Infrared Spectroscopy (FTIR)

FTIR spectra of wasp chitosan were recorded on a FTIR (4100Jasco-Japan) spectrophotometer. The specimen was mixed with KBr. Within 20 scans, the spectral area between 4000 and 400 cm^−1^ was examined at a resolution of 2 cm^−1^.

#### 2.2.2. The Viscosity

The viscosity of wasp chitosan was measured by using rotary torque viscometer performed at a temperature of 22 ± 0.5 °C, and experiments were performed in triplicate according to Kittur et al. 2003 [24]. The volume of the sample was 250 mL, spindle S001, and at 100 rpm. 

### 2.3. Conversion of Wasp Chitosan (WCS) into Wasp Chitosan Nanoparticles (WCSNPs) 

Wasp chitosan nanoparticles (WCSNPs) were prepared by ionic crosslinking of chitosan with trisodium polyphosphate (TPP). Briefly, 0.5 g WCS was dissolved in 100 mL acetic acid 0.025 % (*v*/*v*) by shaking by hand for 5 min. At room temperature (due to high solubility of WSC, there is no need for a magnetic stirrer), the pH was raised to 4.7 with NaOH, then the solution was filtered with a 0.45 μm syringe filter. WCSNPs were produced spontaneously after 33.33 mL of an aqueous tripolyphosphate solution (0.25 percent, *w*/*v*) was added with magnetic stirring. Cooling centrifugation at 10,000 rpm for 15 min was used to purify the nanoparticles. To eliminate sodium hydroxide, supernatants were removed and WCSNPs were thoroughly washed with distilled water. After that, the nanoparticles were redistributed in Milli-Q filtered water [25,26].

### 2.4. Characterization of Wasp Chitosan Nanoparticles (WCSNPs)

Wasp chitosan nanoparticles (WCSNPs) were characterized by FTIR, the average dynamic size, PDI, and zeta potential measurements by Malvern Zetasizer (Malvern Instruments Limited, UK) at 25 °C, and transmission electron microscopy (TEM), viscosity, and stability tests were also done.

#### 2.4.1. Fourier Transform Infrared Spectroscopy (FTIR) 

FTIR spectra of WCSNPs were recorded on FTIR (4100Jasco-Japan) spectrophotometer. Specimens were mixed with KBr. The spectral region between 4000 and 400 cm^−1^ was scanned with a resolution of 2 cm^−1^ within 20 scans.

#### 2.4.2. Droplet Size Distribution

Using a particle size analyzer (Malvern-UK, 4700), the dynamic light scattering (DLS) technique was utilized to quantify the nanoparticle dynamic and surface charge (zeta potential). The NP suspension was diluted 50 times before being examined in Zeta mode. The quantification was carried out at 25 °C, and the zeta potential was provided as the sample’s average of 20 runs.

#### 2.4.3. Transmission Electron Microscopy (TEM)

A high-resolution transmission electron microscope was used to examine the nanoparticles’ morphological properties (TEM, Tecnai G20, FEI, the Netherlands). A droplet of suspension was put on a copper grid coated with carbon film and was not stained (200 mesh). The excess liquid was removed five minutes later by striking the edge of the copper grid with a piece of filter paper. The sample was then air-dried before being analyzed by TEM.

#### 2.4.4. Stability

WCSNPs’ thermodynamic stability was tested by keeping them separate at 24 °C and 4 °C for three months. Furthermore, WCSNPs were centrifuged at 10,000 rpm for 30 min before being examined for creaming, phase separation, or cracking, as described by [25].

### 2.5. Evaluation of the Antimicrobial Activity of WCSNPs

#### 2.5.1. Microbial Strains

Pure cultures of *K. pneumoniae*, *E. coli*, and *P. aeruginosa* that showed the resistance for both ESBLs and carbapenem were obtained from the previously published paper [27,28].

#### 2.5.2. Bacterial Growth Inhibition 

One pure colony per isolate was injected in brain–heart infusion broth (Oxoid) and cultured in a shaker incubator at 37 °C for 4 h. Dilution of bacterial cultures in brain–heart infusion broth at a concentration of 1 × 10^6^ colony-forming units (CFU)/mL was made and detected by plate count. One milliliter of WCSNP solution was combined with one milliliter of each broth containing bacteria from different species, and the tubes were cultured overnight.

Microbial growth at these conditions was compared for each isolate with one of the control samples, which did not include WCSNPs but contained the same volume of sterile distilled water and bacteria-containing broth.

After incubation, 0.1 mL of each tube was plated on the Mueller Hinton agar plate (Oxoid) and incubated 37 °C for 24 h, followed by colony counting.

#### 2.5.3. Percent of Inhibition of Bacterial Growth

The antibacterial activity of the prepared WCSNPs was examined against the carbapenem and ESBL-producing *K. pneumoniae*, *E. coli*, and *P. aeruginosa* and evaluated by calculation of the percentage of growth inhibition. The antibacterial activity was tested at pH < 6 by acidifying with acetic acid (1% *w*/*v*) LB for *K. pneumoniae*, *E. coli*, and *P. aeruginosa*. To prepare the inoculum, all the bacterial cell suspensions were adjusted to 10^6^ CFU/mL [29]. 10 μL of bacterial strain was injected into the previously filled 100 μL of sterile LB broth comprising 96-well plates using the microdilution technique with Luria-Bertani broth (LB) [30].

To assess the antibacterial properties, 100 μL of WCSNPs at starting concentrations of 10 μg/mL were transferred to plates and the increasing concentrations (20, 30, 40, 50, 60, 70, 80, 90, and 100 μg/mL) of the WCSNPs were filled into the corresponding wells. 

The bacterial culture alone (without chitosan) in sterile broth served as a control, and acetic acid controls (LB at pH 5 and pH 6.2) were also examined to assess the potential antibacterial impact of acidification. To guarantee reliable and repeatable findings, all samples were examined in triplicate.

The plate was incubated at 37 degrees Celsius for 24 h. After incubation, the MIC value was obtained by examining the turbidity of the bacterial growth. The MIC value was defined as the amount that inhibited 99% of the examined bacterial growth, and the optical densities (OD) of the plate were measured using a UV–vis spectrophotometer at 570 nm to determine the level of growth inhibition. The experiment was carried out three times, and the percentage of inhibition was calculated as follows: Inhibition (%) = ((Control OD 570 nm) (Test OD 570 nm)/(Control OD 570 nm) × 100.

## 3. Results 

### 3.1. Characterization of Extracted Chitosan

Extracted wasp chitosan was soluble in 0.025% acetic acid, characterized using the FTIR technique, and compared with the wavenumber bands of the wasp chitosan.

#### 3.1.1. Fourier Transform Infrared Spectroscopy (FTIR) of Wasp Chitosan

**Figure 1:**
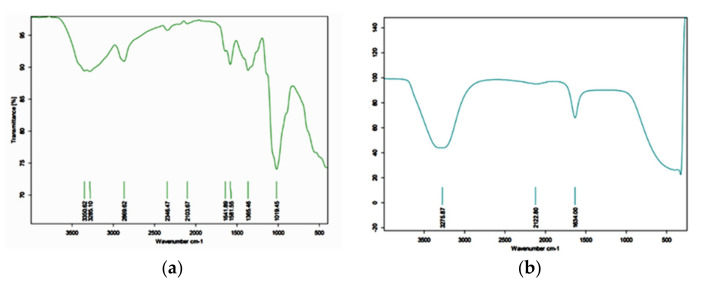
FTIR images of (**a**) WCS; (**b**) WCSNP.

A broad peak centered at 3420 cm^−1^ was attributed to O-H and N-H stretching vibrations, and the peak at 2882.9 cm^−1^ was due to the −CH stretching vibration in −CH and −CH_2_. The FTIR of chitosan showed bands that can be assigned as: 1650 cm^−1^ (−NH bending vibration in −NH_2_), 1424 cm^−1^ (−NH deformation vibration in −NH_2_), 1154 cm^−1^ (−CN stretching vibration), 1061.4 and 1031 cm^−1^ (−CO stretching vibration in −COH), and 897.6 cm^−1^ (−CN stretching vibration (Figure 1a).

#### 3.1.2. Viscosity Analysis

Viscosity analysis of WCS was shown in Table 1 where MW was measured to be about 1990 Da according to [24].

### 3.2. Characterization of Wasp Chitosan Nanoparticles (WCSNPs)

#### 3.2.1. Fourier Transform Infrared Spectroscopy (FTIR)

The FTIR spectrum of WCSNPs exhibited peaks at 3351 and 3258 cm^−1^ endorsed to –NH2 and –OH groups’ stretching vibration. The peaks at 1641 cm^−1^ and 1581 cm^−1^ are attributed to the CONH_2_ and NH_2_ groups, respectively. The peak at 1019 cm^−1^ shows characteristics of the P=O stretching vibration from phosphate groups (Figure 1b). 

#### 3.2.2. Dynamic Light Scattering (DLS) & Polydispersity Index (PDI)

**Figure 2:**
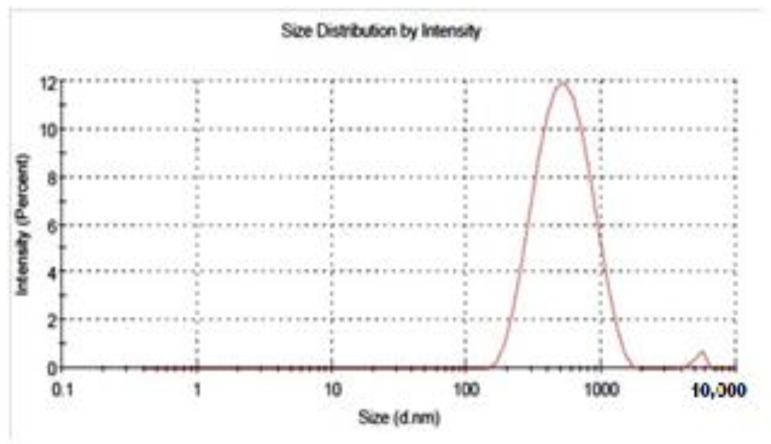
Droplet size distribution and polydispersity index (PDI) of WCSNPs.

The average hydrodynamic diameter of chitosan nanoparticles measured by DLS was found to be 477 nm with a polydispersity index (PDI) of 0.241. This size is higher than that estimated by electron microscopy (Figure 2).

#### 3.2.3. The Surface Charge of the Nanoparticles 

**Figure 3:**
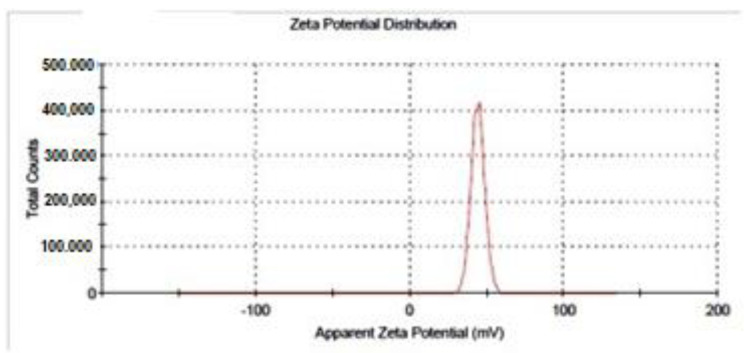
Apparent zeta potential of WCSNPs assessed by Malvern Zetasizer ZS.

In this study, the surface charge was measured by Malvern Zetasizer ZS. The zeta potential was found to be 43.9 ± 4.25 mV, (Figure 3) which was also assessed by Malvern Zetasizer ZS.

#### 3.2.4. Transmission Electron Microscopy (TEM)

**Figure 4:**
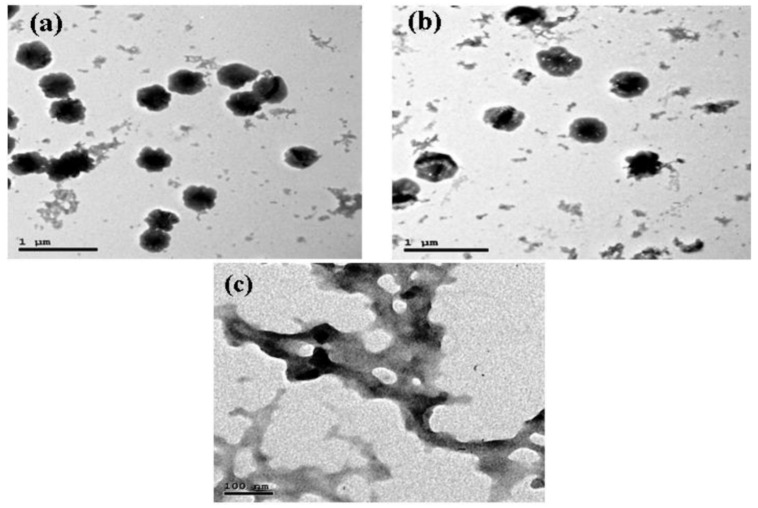
Morphology of chitosan nanoparticles visualized by transmission electron microscopy. The scale bars correspond to 1 μm (**a**,**b**), 100 nm (**c**).

TEM can validate the size and surface shape of nanoparticles. The TEM image of chitosan nanoparticles prepared at pH 4.7 revealed the formation of the particles with an average size of 200–280 nm with narrow size distribution (Figure 4).

### 3.3. Stability 

Results showed that after 3 months, WCSNPs were not observed during creaming, phase separation, or cracking.

### 3.4. Evaluation of the Antimicrobial Activity of WCSNPs

The produced WCSNPs showed growth inhibition effectiveness against all tested strains, and for all tested isolates, the bacterial count was lower than the initial count prior to incubation.

**Figure 5:**
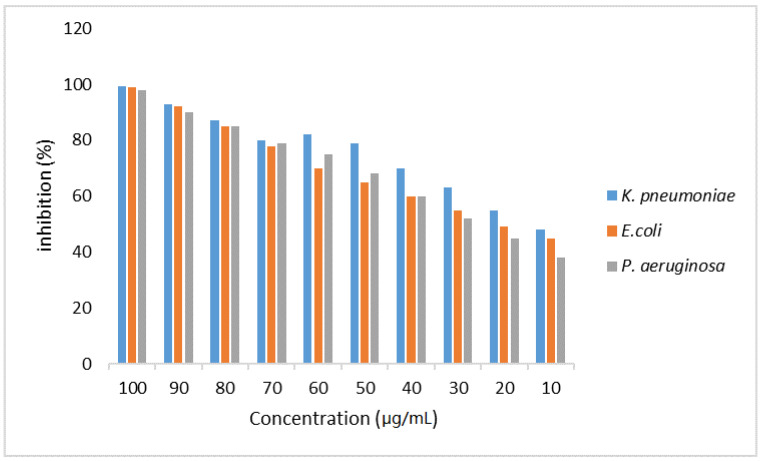
The percent of bacterial growth inhibition of WCSNPs against positive *K. pneumoniae*, *E coli*, and *P. aeruginosa* at concentrations from 10 μg/mL to 100 μg/mL.

The inhibition percent of WCSNPs against *K. pneumoniae*, *E coli*, and *P. aeruginosa* at concentrations 10 μg/mL and 100 μg/mL is displayed in Figure 5.

After 24 h incubation, the maximum growth inhibitions of *K. pneumoniae*, *E. coli*, and *P. aeruginosa* were found to be 99.4%, 98.9%, and 97.8% at 100 μg/mL, respectively. On the other hand, the inhibition percentages were 48%, 45%, and 38% against *K. pneumoniae*, *E. coli*, and *P. aeruginosa* at 10 μg/mL, respectively. It was found that while the concentration of WCSNPs increases, the growth of extended-spectrum beta-lactamase- and carbapenemase-producing isolates decreases.

## 4. Discussion

The disparity between the establishment and dissemination of resistance mechanisms and the discovery of novel antimicrobial compounds is a serious public health problem. Therefore, it is critical to consider novel approaches for combating resistance, such as the discovery of new compounds as well as the application of nanotechnology as an intriguing alternative to traditional antibiotic research methods [31,32,33,34]. 

Chitosan is a non-toxic, biocompatible, and biodegradable linear cationic biopolymer produced from the alkaline deacetylation of chitin [35]. The original source of chitosan, crustacean waste, is no longer sustainable, and fungi, a potential substitute, have yet to be exploited on a significant basis. On the alternative, the rearing of bio-converting insects, as well as the recycling of insect biomass, represent a waste stream of facilities that may be utilized for several applications. Because of its identical commercial qualities, insect chitosan has been used in the same industries as crustacean biopolymers [8]. Our source of isolation is wasp chitosan, extracted from *Vespa orientalis*, which is characterized by its good solubility in 0.025% acetic acid due to the high degree of deacetylation (92–93%) that we recorded in our previous study [8]. The DD of insect-derived chitosan varies from 60 to 98% depending on the deacetylation settings used and is completely comparable to commercial chitosan made from crustaceans [36,37,38]. However, DD values vary greatly depending on both the chitosan supply and the deacetylation process used [39,40].

The molecular weight (MW) of chitosan also plays an important role in defining its effectiveness in a variety of applications. The MW was determined to be about 1990 Da since it has a significant impact on its biological activity [41,42]. In general, the MW of insect-derived chitosan ranges from 26 to 300 kDa [38], lower than that of commercial chitosan (100–1000 kDa) [43], so the good solubility in a low concentration of acetic acid is attributed to its low molecular weight; in addition, it is related to greater antimicrobial activity [41,42].

However, low-molecular-weight chitosan is generally obtained from commercial chitosan by chemical, physical, or enzymatic methods, and has much higher solubility and stability than commercial chitosan, so obtaining such a source of low MW without any process is an excellent outcome, especially since the choice of type of chitosan hydrolysis remains a challenge. Obviously, several factors impact the process, including yield, cost, and the qualities of the hydrolyzed product. Decomposition, such as with chemicals, has downsides, such as severe hydrolysis conditions and limited productivity. Furthermore, the physical breakdown of chitosan necessitates the use of specialized equipment, and the resultant MW cannot be controlled.

In the current investigation, FTIR analysis of wasp chitosan revealed the band spectrums of various bonds, which are consistent with [8] and others [43,44]. The ionic interaction between the positively charged NH3+ groups of chitosan and phosphate groups and the negatively charged phosphate groups (P3O105-) of TPP was ascribed to the changes seen in the FTIR spectra of WCSNPs in the peaks at 1641 cm^−1^ and 1581 cm^−1^. The peak at 1019 cm^−1^ exhibits P=O stretching vibration properties from phosphate groups. In the earlier investigation, similar results were observed of the production of chitosan nanoparticles treated with TPP [45,46].

Notably, the average hydrodynamic diameter of chitosan nanoparticles measured by DLS was found to be 477 nm with a polydispersity index (PDI) of 0.241. This size is more than that estimated by electron microscopy, and it is due to the significant swelling capacity of chitosan nanoparticles. DLS provides the particle’s hydrodynamic radius, whereas SEM provides an estimate of the projected area diameter. A tiny electric dipole layer of the solvent sticks to the surface of a dispersed particle in DLS when it travels through a liquid medium. This layer has an impact on particle mobility; [47,48] discovered the best CS/TPP *w*/*w* ratio to be 4:1, which yielded nanoparticles with diameters of 340 nm, but for other CS/TPP ratios, the size of the nanoparticles increased.

The zeta potential is an important metric for aqueous nanosuspensions’ stability. A zeta potential of ±30 mV is required as the minimum for a physically stable nanosuspension sustained purely by electrostatic repulsion [48]. All this evidence revealed that the chitosan nanoparticles prepared here were stable.

Visualization under TEM can establish the size and surface shape of nanoparticles. The TEM picture of chitosan nanoparticles synthesized at pH 4.7 indicated that the particles formed with an average size of 200–280 nm and a narrow size distribution. It is known that the average chitosan/TPP particle size is affected by a variety of variables, including the mixing technique [6,49,50], chitosan and TPP concentration ratio [51,52], chitosan degree of deacetylation (DD) [53,54], temperature [11], ionic strength, and pH [51,55,56]. Particle size in each of these cases is determined by two factors: (1) their swelling properties, which are affected by pH [16,18], ionic strength [13], and TPP: chitosan ratio [47], and (2) their aggregation number, which is the number of aggregated chitosan chains in an average chitosan/TPP particle [55,57]. This aggregation number is regulated kinetically and represents the TPP-mediated synthesis and aggregation of primary chitosan/TPP nanoparticles into higher-order structures [58]. Because the charge densities of chitosan and TPP are pH-dependent, pH is a crucial factor influencing the ionic interaction of TPP and chitosan.

According to [25], no WCSNPs were found during creaming, phase separation, or cracking. The chitosan–TPP nanoparticle colloidal system is thermodynamically unstable in general due to the high surface energy associated with the nanoscale dimensions, especially under unfavorable solution pH conditions and at high particle concentrations.

The surface charge of nanoparticles is an essential component in the stability of nanoparticulate solutions. Recently, the role of positive nanoparticle charge in cytoplasmic trafficking was investigated, and it was shown that positive nanoparticles may connect to anionic microtubules or molecular motor proteins and migrate towards the cell nucleus via the cytoskeletal network [59].

In this study, the surface charge was measured by Malvern Zetasizer ZS. The zeta potential was found to be 43.9 ± 4.25 mV (Figure 3), and this value agrees with +47.2 mV obtained by [54]. The high positive value of zeta potential suggests the chitosan nanoparticles are stable and will be able to bind with the cell membrane and increase the cellular uptake. It was observed that pH, the chitosan/TPP ratio, and the N/P ratio had significant effects on the surface charge of the nanoparticles.

Biopolymers can be used efficiently as carrier molecules. They offer significant promise for utilizing WCSNPs as a nano-carrier system for encapsulating cefazolin, as well as their prospective application as nano-antibiotics for treating resistant bacterial infections caused by major Gram-negative pathogens [60].

The overuse of antimicrobials in human and veterinary medicine is the primary cause of an ongoing rise in antibiotic resistance. A global priority list of pathogenic bacteria that are resistant to antibacterial agents was issued by the World Health Organization (WHO), and it included several *Enterobacteriaceae* for whom novel antibiotics are urgently required [61]. The prevalence of MDR zoonotic pathogens such as *K. pneumoniae*, *E. coli* and *P. aeruginosa* has been significantly increasing over the last decade in the human population and causing severe clinical outcomes. Recently, extended-spectrum β-lactamase (ESBL)- and carbapenemase-producing bacteria have spread around the world in both humans and animals, of which the majority of ESBL- and carbapenemase-resistant genes are found on mobile genetic components such plasmids, causing a threat to public health [2,62].

Therefore, in this study, the antibacterial activities of WCSNPs against positive *K. pneumoniae*, *E coli*, and *P. aeruginosa* were investigated. The results indicated that the percent of growth inhibition of the synthesized nanomaterials can credibly help fight the growth of ESBL- and carbapenem-resistant *K. pneumoniae*, *E coli*, and *P. aeruginosa* upon increasing concentration. WCSNPs showed antibacterial activity against the tested isolates, even at 10 μg/mL. 

This finding is consistent with the findings of [60], in which the authors prepared antibiotic-loaded nanoparticles and tested them against multidrug-resistant *K. pneumoniae*, *P. aeruginosa*, and ESBL-producing *E. coli* and affirmed that chitosan nanoparticles can be a prospective carrier system for targeting antibiotic-resistant microorganisms due to their excellent antimicrobial activity and biocompatibility.

Several studies [63,64,65,66,67] found that the antibacterial activity of CS and CSNPs is substantially influenced by their MW and DA. Both MW and DA affect the antibacterial activity of CS and CSNPs, although MW has a greater impact than DA [65,68]. The MW of WCS allowed for satisfactory changes in the antibacterial ability of the synthesized NPs, similar to what [63] reported. They concluded that CSNPs with low and medium MWs may effectively suppress pathogen development and that the inhibitory capacity can be regulated by pH and MW, as an increase in MW of CS leads to a rise in size and a drop in the zeta potential, lowering antibacterial activity. A reduction in the MW of CS produced smaller-sized particles and increased zeta potential. Therefore, greater zeta potential facilitates NP attachment and communication with the bacterial cell membrane. 

The alteration in MW and particle size/zeta potential enabled simple adjustment of the physicochemical parameters of the NPs. Other investigations have indicated that the size and zeta potential of CSNPs, as well as their efficacy against bacterial species, are highly dependent on their MW [51,69,70]. Moreover, another study found that CSNPs with greater MW had lower antibacterial activity. This shows that antibacterial activity and efficiency are highly reliant on the bacterial species and CS source.

In conclusion, insects are the most promising source of chitosan as an alternative to crustaceans, especially on an industrial scale after they were found to have their unique properties. The antibacterial activity of the prepared WCSNPs was examined against extended-spectrum beta-lactamase (ESBL)- and carbapenemase-producing *Klebsiella pneumoniae*, *Escherichia coli*, and *Pseudomonas aeruginosa*.

The created WCSNPs effectively inhibited the growth of all tested strains. It was discovered that raising the concentration of WCSNPs inhibits the development of isolates that produce extended-spectrum beta-lactamases and carbapenemase.

Our findings demonstrated that WCSNPs can be a good carrier system for targeting antibiotic-resistant microorganisms because of their excellent antibacterial activity and biocompatibility.

## Figures and Tables

**Table 1:** Viscosity analysis of WCS.

Container and Volume	Sample Volume (mL)	Temperature °C	Spindle	RPM	Torque%	Average c.P	Mwt
Beaker 250	250	22	S001	100	13.8	13.8 ± 0.057	1990 Da

## Data Availability

All data generated or analyzed during this study are included in this published article.

## Sample Availability

Samples of the compounds are not available from the authors.
